# Chronological development of in-patient oncology in times of COVID-19: a retrospective analysis of hospitalized oncology and COVID-19 patients of a German University Hospital

**DOI:** 10.1007/s00432-022-04044-8

**Published:** 2022-06-30

**Authors:** Sebastian Griewing, Uwe Wagner, Michael Lingenfelder, Rebecca Fischer, Matthias Kalder

**Affiliations:** 1 Department of Gynecology and Obstetrics, University Hospital Marburg, Philipps-University Marburg, Baldingerstraße, 35043 Marburg, Germany; 2 Chair of General Business Administration, Institute for Health Care Management e.V., Philipps-University Marburg, Universitätsstraße 24, 35037 Marburg, Germany

**Keywords:** COVID-19, University hospital, Oncology, Lockdown, Virus variants

## Abstract

**Purpose:**

The goal of this study is to examine the chronological development of hospitalized oncology and COVID-19 patients, and compare effects on oncology sub-disciplines for pre-pandemic (2017–19) and pandemic (2020–21) years in the setting of a German university maximum care provider.

**Methods:**

Data were retrospectively retrieved from the hospital performance controlling system for patient collectives with oncological main (n_Onco_) and COVID-19 secondary diagnosis (n_COVID-19_). Data analysis is based on descriptive statistical assessment.

**Results:**

The oncology patient collective (n_Onco_ = 27,919) shows a decrease of hospitalized patients for the whole pandemic (− 4% for 2020 and − 2,5% for 2021 to 2019). The number of hospitalized COVID-19 patients increases from first to second pandemic year by + 106.71% (n_COVID-19_ = 868). Maximum decline in monthly hospitalized oncology patients amounts to − 19% (May 2020) during the first and − 21% (December 2020) during the second lockdown. Relative monthly hospitalization levels of oncology patients reverted to pre-pandemic levels from February 2021 onwards.

**Conclusion:**

The results confirm a decline in hospitalized oncology patients for the entire pandemic in the setting of a maximum care provider. Imposed lockdown and contact restrictions, rising COVID-19 case numbers, as well as discovery of new virus variants have a negative impact on hospitalized treated oncological patients.

## Introduction

Since the onset of the rapidly advancing pandemic, spread of COVID-19 in the spring of 2020 society worldwide has been characterized by significant restrictions. Over the course of the last 2 years, the Federal Republic of Germany has registered about 7.109.182 confirmed COVID-19 cases (data from December 30th of 2021) (Robert Koch Institut 2021). The state of Hesse confirmed 477,279 COVID-19 cases with 8548 fatal outcomes leaving a state mortality rate of 1.8% on December 30th 2021 according to the Robert Koch Institute, the central national institution in the field of disease surveillance and prevention in Germany (Robert Koch Institut 2021). A variety of measures have been enforced politically restricting work and social life in a counterplay of recurrent lockdowns and gradual easing attempts. Internationally, the care of acute and chronic diseases has experienced noxious effects due to the pandemic (Czeisler et al. 2020; Lazzerini et al. 2020; Tangcharoensathien et al. 2021). To guarantee planning security and prevent critical capacity overload for the health care system, strong political influence was exerted on the medical care structure and organization which made regular service provision in German hospitals almost impossible at times. Oncology has widely been discussed as the medical field being particularly vulnerable to negative impact of the pandemic on the care situation (Alagoz et al. 2021; Andrew et al. 2021; De Luca et al. 2022; Earnshaw et al. 2020; Erdmann et al. 2021; Gurney et al. 2021; Jacob et al. 2021; Kuzuu et al. 2021; Patt et al. 2020; Peacock et al. 2021; Piontek et al. 2021; Reichardt et al. 2021; Ruiz-Medina et al. 2021; Stang et al. 2021; Tsibulak et al. 2020; Vardhanabhuti and Ng 2021; Voigtländer et al. 2021). In Germany, studies conducted in various states, e.g., Saxony (Piontek et al. 2021), North Rhine-Westphalia (Stang et al. 2021), or Bavaria (Voigtländer et al. 2021), have confirmed a decline of cancer cases during different time periods of the pandemic and on national scale studies have confirmed a decrease in oncological cases in general and specialized practices in Germany for April 2020–March 2021 and March–May 2020 (Jacob et al. 2021, 2022). The current literature focusses on parts of the pandemic and leaves out the perspective of an in-patient oncological maximum care provider.

This was an occasion to examine and compare the entire pandemic period of 2020 and 2021 with the previous years of 2017–2019 as a university maximum service provider of the state of Hesse with regard to the development of hospitalized oncology and COVID-19 patients, whether oncological sub-disciplines differ in terms of absolute and relative change of case count and how the monthly relative case development of admitted oncological patients relates to national lockdown and contact measures or to the discovery to new COVID-19 virus variants, e.g., delta and omicron virus variants.

## Methods

### Data generation

The data for the present analysis were retrospectively generated for the time period of 1st January 2017–31st December 2021 and retrieved from the hospital performance controlling program QlikView^®^ of Marburg University Hospital, which records all medical, nursing, and equipment services coded in the hospital information system. Data were collected for all patients with a main oncology n_Onco_ (ICD C-diagnosis) and a secondary COVID-19 diagnosis n_COVID-19_ (ICD-code “U07.1 COVID-19, virus identified”). Furthermore, the oncological patient collective was divided in ICD-based groups for oncology sub-disciplines depicted in Table 3 and the statistical protocol was repeated. The data were fully anonymized before analysis.

### Statistical methods

The evaluation is exclusively based on methods of descriptive statistics. The focus of the study lies on the analysis of the patient collective regarding age and gender distribution, the monthly and yearly relative development of the overall number of hospitalized patients, as well as the recorded main and secondary ICD diagnoses.

## Results

### Descriptive analysis of the patient collectives

A total of n_Onco_ = 27,919 oncology patients have been hospitalized at Marburg University Hospital within the observation period. The data show a yearly relative increase in total case number for pre-pandemic years (+ 3% 2017–18, + 6% 2018–19) followed by decrease of − 4% (2019–20) for the first and an increase of + 1% (2020–21; − 2,5% 2019–21) for the second pandemic year. The oncology patient collective is divided in 45.4% female and 54.5% male with an average age of 64 and 66.1, respectively (*n* = 9 cases with unspecified gender). Regarding the in-patiently treated COVID-19 patient collective, a total of n_COVID-19_ = 868 patients were analyzed leaving a relative increase of + 106.71% (2020–21) between the first and second pandemic year. The gender distribution divides in 42.2% female and 57.8% male with a mean age of 63.2 and 62, respectively. Absolute figures and the ten most common main diagnoses are illustrated in Tables 1 and 2.

**Table 1:** Absolute monthly hospitalized oncology and COVID-19 patients of Marburg University Hospital, 2017–21

	Absolute monthly hospitalized oncology patients (2017–2021)					Absolute monthly hospitalized COVID-19 patients (2020–21)	
	2017	2018	2019	2020	2021	2020	2021
Jan	484	538	531	555	419	0	103
Feb	417	411	437	461	474	0	67
Mar	476	393	484	498	488	12	68
Apr	404	465	498	426	442	15	97
May	480	425	518	422	478	4	40
Jun	419	448	443	478	446	1	11
Jul	451	495	568	492	468	7	3
Aug	461	506	462	498	529	1	13
Sep	439	438	474	444	507	6	35
Oct	436	493	495	474	506	47	23
Nov	493	484	504	459	492	94	53
Dec	390	390	400	395	418	96	72
Sum	5.350	5.486	5.814	5.602	5.667	283	585
Relative development to previous year, in %		3%	6%	− 4%	1%		107%

**Table 2:** Most common main diagnoses of hospitalized oncology and COVID-19 patients of Marburg University Hospital, 2017–21

	Oncological main diagnosis, 2017–21	Total cases	Share in %	Σ %
	Sum	27.919	100	31.14
1	C61 Malignant neoplasm of the prostate gland	1.460	5.23	5.23
2	C50.4 Malignant neoplasm: upper outer quadrant of mammary gland	1.222	4.38	9.61
3	C44.3 Other malignant neoplasms: Skin of other and unspecified parts of the face	1.196	4.28	13.89
4	C20 Malignant neoplasm of the rectum	805	2.88	16.77
5	C79.3 Secondary malignant neoplasm of the brain and meninges	731	2.62	19.39
5	C83.3 Diffuse large B-cell lymphoma	731	2.62	22.01
7	C34.1 Malignant neoplasm: upper lobe (-bronchus)	707	2.53	24.54
8	C67.8 Malignant neoplasm: Urinary bladder, overlapping several sections	628	2.25	26.79
9	C90.00 Multiple myeloma: without indication of complete remission	621	2.22	29.02
10	CC92.00 Acute myeloblastic leukemia [AML]: without indication of complete remission	594	2.13	31.14

	Main diagnosis of hospitalized patients with COVID-19 infection, 2020–21	Total cases	Share in %	Σ %
	Sum	868	100.00	72.12
1	J12.8 Pneumonia due to other viruses	528	60.83	60.83
2	B34.2 Infection due to coronaviruses of unspecified localizationn	17	1.96	62.79
3	J22 Acute lower respiratory tract infection, unspecified	12	1.38	64.17
4	A08.3 Enteritis due to other viruses	7	0.81	64.98
4	C83.3 Diffuse large B-cell lymphoma	7	0.81	65.78
4	S72.01 Femoral neck fracture: intracapsular	7	0.81	66.59
7	A09.0 Other and unspecified gastroenteritis and colitis of infectious origin	6	0.69	67.28
7	I50.14 Left heart failure: with symptoms at rest	6	0.69%	67.97
7	I63.4 Cerebral infarction due to embolism of cerebral arteries	6	0.69%	68.66
10	B34.2 Infection due to coronaviruses of unspecified location	5	0.58%	69.24
10	J12.9 Viral pneumonia, unspecified	5	0.58%	69.82
10	N39.0 Urinary tract infection, localization unspecified	5	0.58%	70.39
10	O99.5 Diseases of the respiratory system complicating pregnancy, childbirth, and puerperium	5	0.58%	70.97
10	R06.0 Dyspnea	5	0.58%	71.54
10	R53 Unwellness and fatigue	5	0.58%	72.12

### Yearly absolute and relative development of oncology sub-disciplines (2017–21)

The yearly relative development and absolute patient numbers of the oncology sub-disciplines for pre-pandemic (2017–19) as well as pandemic years (2020–21) in comparison to the pre-pandemic baseline of 2019 are depicted in Table 3. Furthermore, the overall relative share of the n_onco_ = 27,919 cases for whole observation period was identified for each oncological sub-discipline.

**Table 3:** Yearly relative and absolute patient number development of oncology sub-disciplines (2017–21)

ICD sub-groups	Absolute patient numbers per sub-group, 2017–2021					Yearly relative change in hospitalized patients per sub-group in %				Share of overall case count 2017–2021, in %
	17	18	19	20	21	17 to 18	18 to 19	19 to 20	19 to 21	
C00-C14 Malignant neoplasms of the lip, oral cavity and pharynx	266	306	285	266	324	15%	− 7%	− 7%	14%	5%
C15-C26 Malignant neoplasms of the digestive organs	930	935	997	973	937	1%	7%	− 2%	− 6%	16%
C30-39 Malignant neoplasms of the respiratory organs and other intrathoracic organs	504	555	554	512	558	10%	0%	− 8%	1%	9%
C40-41 Malignant neoplasms of the bone and articular cartilage	31	30	20	58	49	− 3%	− 33%	190%	145%	1%
C43-C44 Melanoma and other malignant neoplasms of the skin	645	668	647	693	669	4%	− 3%	7%	3%	11%
C45-C49 Malignant neoplasms of the mesothelial tissue and soft tissue	71	57	56	72	70	− 20%	− 2%	29%	25%	1%
C50 Malignant neoplasms of the mammary gland	453	473	506	475	516	4%	7%	-6%	2%	8%
C51-C58 Malignant neoplasms of the female genital organ	326	317	344	318	290	− 3%	9%	− 8%	− 16%	6%
C60-C63 Malignant neoplasms of the male genital organs	359	338	420	391	351	− 6%	24%	− 7%	− 16%	6%
C64-68 Malignant neoplasms of the urinary organs	340	393	398	339	326	16%	1%	− 15%	− 18%	6%
C69-C72 Malignant neoplasms of the eye, brain and other parts of the central nervous system	134	144	133	140	130	7%	− 8%	5%	− 2%	2%
C73-C75 Malignant neoplasms of the thyroid and other endocrine glands	222	193	159	157	138	− 13%	− 18%	− 1%	− 13%	3%
C76-C80 Malignant neoplasms of vaguely defined, secondary and unspecified locations	688	677	712	657	715	− 2%	5%	− 8%	0%	12%
C81-C96 Malignant neoplasms of lymphoid, hematopoietic and related tissues, established or suspected as primary	634	623	762	766	781	− 2%	22%	1%	2%	12%

### Monthly relative case development of the oncology and COVID-19 patient collectives (2019–21)

The monthly case count of all in-patient cases with an oncological main diagnosis admitted at the Marburg University Hospital in 2019 was used as a pre-pandemic baseline to visualize the relative monthly change in comparison to the pandemic years of 2020 and 2021. The illustration is combined with dates of lockdown and contact restriction measures taken by the Government of the Federal Republic of Germany and dates of the first national cases of COVID-19 delta and omicron virus variants.

## Discussion

### Main findings

The analysis illustrates a worrisome chronological development of in-patient oncology cases for the catchment area of Marburg University Hospital. The data confirm the national and international recognized decline in oncological cases for the setting of a maximum service provider for the entire pandemic time of 2020 and 2021, and show differences for oncological sub-disciplines (Andrew et al. 2021; De Luca et al. 2022; Erdmann et al. 2021; Gurney et al. 2021; Patt et al. 2020; Peacock et al. 2021; Piontek et al. 2021; Reichardt et al. 2021; Ruiz-Medina et al. 2021; Stang et al. 2021; Tsibulak et al. 2020; Vardhanabhuti and Ng 2021; Voigtländer et al. 2021). After 3 years of consecutive increase in oncological case count, that predominantly connects to the successive closure of regional peripheral care providers and the vice versa expansion of the oncological care network, the pandemic has stirred up oncological care in the catchment area of Marburg University Hospital.

### Interpretation of findings

#### Comparison of oncological sub-disciplines

Worrisome, sub-groups with a high share of overall case count tend to realize a relative decline in the first pandemic year that intensifies for the second (i.e., − 15% in 2020 and − 18% in 2021 for uro-oncology C64-68; − 2% and − 6% for gastrointestinal oncology C15-C26; − 6% and − 18% for gyne-oncology of inner female genitals C51-C58). The extent of patient number decline for C60-63 and C64-C58 sub-groups could fraudulently be intensified by the departure of two specialized urologists for 2021. Other prominent sub-disciplines overcome the pandemic effects in the second year of observation (i.e., − 6% and + 2% for breast cancer C50; − 8% and + 1% for respiratory and thoracic cancer C30-39*)* or seem to be not affected at all (i.e., + 7% and + 3% for malignant skin cancer C43-C44; + 1% and + 2% for haemato- and lymphoid oncology C81-C96)*.* International findings are coherent with these developments and confirm this noxious but divergent developments (Jacob et al. 2022; Kuzuu et al. 2021; Monroy‐Iglesias et al. 2022). Changes in sub-disciplines with small overall share (≤ 5% of overall oncology cases) tend to be biased by structural changes, managerial decisions, or changes in assigning out-patient physicians and practices, and display counterintuitive development (i.e., + 190% and 145% for bone and cartilage cancer C40-41, + 29% and + 25% for mesothelial cancer C45-C49). The contradicting increase for C40-41 for instance is likely affected by the external factor of an ownership exchange of an assigning local practice. Further managerial and structural biases can be ruled out to a large extent in case of Marburg University Hospital. The comparison of German out-patient and in-patient case numbers suggests that especially cancer types with out-patient screening programs (in Germany, e.g., skin, cervical, breast, colorectal, and prostate cancer) are effected by the pandemic leading to an expected increase of cancer stage upon primary disease diagnosis (Mayo et al. 2021).

#### Comparison of chronological development of oncology and COVID-19 patient collectives

**Fig. 1:**
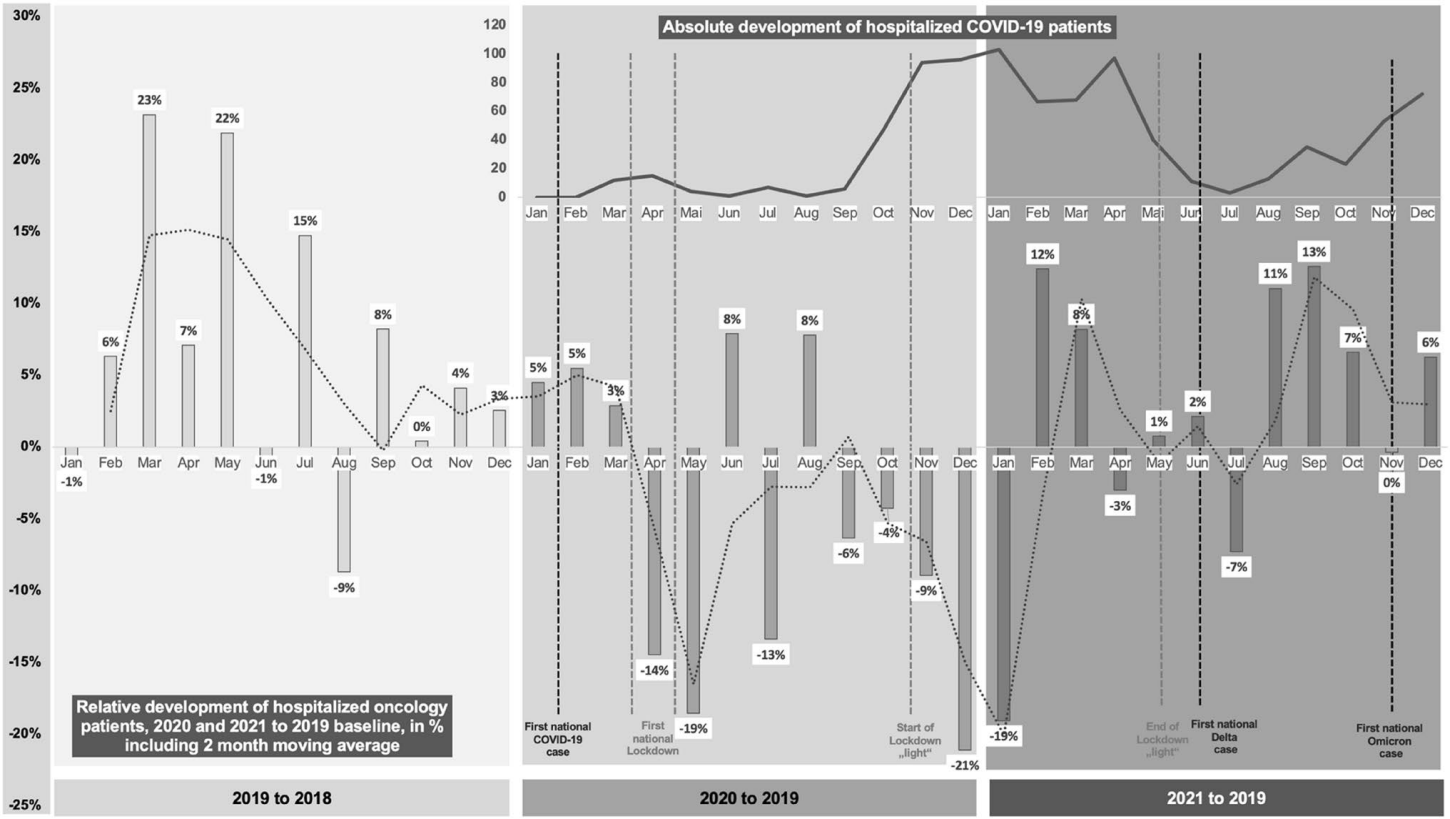
Monthly relative development of hospitalized oncology and COVID-19 patients (2019–2021)

On January 27th of 2020, the first national COVID-19 case was registered in Germany. Due to the swift case development, the government of the Federal Republic of Germany initiated a “hard” lockdown with complete contact restrictions on March 22nd. Decision on first relaxations of contact measures was taken on May 3rd of 2020. During summer, only minor restrictions were maintained and the government reacted to a second accelerated COVID-19 case development by imposing a second lockdown “light” in early November including softer contact measures than during the first “hard” lockdown. Following the first national lockdown, Fig. 1 depicts a fierce decline in monthly relative case development of hospitalized oncology patients with a negative maximum of − 19% in May 2020 for Marburg University Hospital. The months of June and August display the only relative increase for the pandemic part of 2020, although these months realized a relative increase for 2019 and, therefore, we expect the data to be biased to external factors not mirroring the real relative development. As such, the decrease of monthly relative case development of oncological cases worsens with imposition of the lockdown “light” in early November reaching another negative maximum of − 21% in December 2020. As the national vaccination program is initiated in January, it also constitutes the last month of heavy relative decline in regional oncological cases leading to a phase of stabilization and oncological case growth. From February 2021 onwards, the registered monthly hospitalizations nearly fully revert to pre-pandemic levels. Figure 1 supports the second wave of COVID-19 cases to be far more intense in terms of in-patiently treated COVID-19. In contrast to other states in Germany, the Hessian state government did not actively interfere with hospital management by imposing a necessary amount of blocked beds for COVID-19 treatment, but provided accurate prediction of bed demand and, therefore, in Hesse proactive bed management for COVID-19 leaving no major discrepancies between used and blocked beds. An international study for 15 tumor types in 61 countries confirms a fragility of cancer surgery to lockdowns with one in seven patients in “hard” lockdowns not undergoing planned surgery for the first 3 months from local emergence of COVID-19 (Glasbey et al. 2021). While most of the literature only focusses on the first national “hard” lockdown with complete contact restrictions, the hereby presented study not only confirms the development during the first national “hard” lockdown for a German maximum service provider, but beyond that enables a comparison with the second “soft” lockdown. Thus, the second so-called German “lockdown light” lasting for a period of more than three times as long as the first one and that did not include complete contact restrictions, but, e.g., limitations to the certain amount of group sizes that varied according to incidence, compares to the first full lockdown in terms of decline of in-patient oncological cases. Interestingly, national number of COVID-19 cases as well as the registered in-patient of Marburg University Hospital clearly surpass the levels of the first “hard” lockdown, but the chronological development of in-patient oncological cases is similar.

While higher morbidity and mortality for the delta variant (AlQahtani et al. 2021; He et al. 2021; Hussey et al. 2021; Khedar et al. 2021) as well as faster spread due to higher contagiousness for the omicron variant (Grabowski et al. 2022; Pulliam et al. 2021) have widely been discussed, little is known about the consequences for oncological care. Furthermore, the swift increase of omicron variant is combined with a lower vaccination efficacy (Burki 2022) that presents to be even worse in cancer patients (Zeng 2021). To our knowledge, no study has investigated the effect of the discovery of new COVID-19 virus variants on in-patient oncological cases development. In June 2021, the first official delta virus variant case of COVID-19 was reported. While Marburg University Hospital realizes a relative increase in monthly relative case development of regional oncological cases for June 2021, it is followed by fierce decline of − 7% in July. The first omicron virus variant cases were officially reported in November 2021 and monthly regional oncological cases were identical for the same month in comparison to the 2019 baseline, while October and December realized a relative increase of + 7% and + 6%. The data suggest that the discovery of new virus variants of COVID-19 may have a negative effect on the monthly relative case development of in-patient oncological cases, although the impact presents to be far more short-term and lower in terms of intensity in comparison to the lockdown and contact measures.

### Limitations

The presented analysis is based on the data of a single regional maximum service supplier of the state of Hesse in Germany, the Marburg University Hospital. Therefore, the transfer of the proposed findings is limited as the data are mainly valid for rural care. Effects of external variables cannot be ruled out based on the presented analysis and an expansion of the analysis to urban areas and multicentric data comparison would be favorable.

### Conclusion

The presented analysis examines and compares the pandemic with the previous pre-pandemic years regarding the chronological development of the in-patient oncological care of Marburg University Hospital. Coherent to international studies, the number of overall cancer cases has declined in the observation period and the analysis suggests that the negative impact on hospitalized oncology patients diverges between sub-disciplines. The findings suggest imposed “hard” and “soft” lockdown and contact restrictions as well as discovery of new virus variants to have a negative impact on in-patiently treated oncology patients. Further research needs to confirm or invalidate these findings in other settings, while it is of utmost importance to measure the effects of lockdown and contact measures as well as discovery of new virus variants to be able to proactively act against a negative impact on cancer care and prohibit an increase in cancer stage upon disease onset.

## Data Availability

The datasets generated during and analyzed during the current study are available from the corresponding author on reasonable request.
